# Multi-contrast laser endoscopy for in vivo gastrointestinal imaging

**Published:** 2025-05-15

**Authors:** Taylor L. Bobrow, Mayank Golhar, Suchapa Arayakarnkul, Anthony A. Song, Saowanee Ngamruengphong, Nicholas J. Durr

**Affiliations:** Department of Biomedical Engineering Johns Hopkins University, Baltimore, MD 21218; Department of Biomedical Engineering Johns Hopkins University, Baltimore, MD 21218; Division of Gastroenterology and Hepatology Johns Hopkins Hospital Baltimore, MD 21287; Department of Biomedical Engineering Johns Hopkins University, Baltimore, MD 21218; Division of Gastroenterology and Hepatology Johns Hopkins Hospital Baltimore, MD 21287; Department of Biomedical Engineering Johns Hopkins University, Baltimore, MD 21218

**Keywords:** colonoscopy, colorectal cancer, endoscopy, laser speckle contrast imaging, multispectral imaging, photometric stereo, topography, tissue oxygen saturation

## Abstract

White light endoscopy is the clinical gold standard for detecting diseases in the gastrointestinal tract. Most applications involve identifying visual abnormalities in tissue color, texture, and shape. Unfortunately, the contrast of these features is often subtle, causing many clinically relevant cases to go undetected. To overcome this challenge, we introduce Multi-contrast Laser Endoscopy (MLE): a platform for widefield clinical imaging with rapidly tunable spectral, coherent, and directional illumination. We demonstrate three capabilities of MLE: enhancing tissue chromophore contrast with multispectral diffuse reflectance, quantifying blood flow using laser speckle contrast imaging, and characterizing mucosal topography using photometric stereo. We validate MLE with benchtop models, then demonstrate MLE *in vivo* during clinical colonoscopies. MLE images from 31 polyps demonstrate an approximate three-fold improvement in contrast and a five-fold improvement in color difference compared to white light and narrow band imaging. With the ability to reveal multiple complementary types of tissue contrast while seamlessly integrating into the clinical environment, MLE shows promise as an investigative tool to improve gastrointestinal imaging.

## Introduction

Flexible endoscopy is a foundational tool for the screening, treatment, and longitudinal assessment of many gastrointestinal disorders, including inflammation, early-stage dysplasia, and cancer. The standard of care is high-definition (HD) white light endoscopy (WLE), which acquires high-resolution (~1 megapixel) widefield images with a red-green-blue (RGB) camera sensor and a broadband illumination source. Gastroenterologists examine endoscopic videos in real time for differences in mucosal color, texture, and shape to identify regions of tissue with abnormalities. Unfortunately, the differences between normal and diseased tissue are often subtle when visualized with WLE. For example, in colonoscopy screening for colorectal cancer (CRC), precancerous adenomas are frequently missed (26%) [1], reducing its protective value against CRC-associated mortality (<50% reduction) [2, 3, 4].

The limited contrast of WLE has motivated the development of complementary optical imaging modalities to improve the detection of abnormal tissues. For example, narrow band imaging (NBI) illuminates tissue with narrow spectral bands of light centered at 415 nm and 540 nm [5]. These colors correspond to spectral absorption peaks of hemoglobin and are rapidly attenuated in tissues, resulting in the enhancement of vascular contrast and the reduction of deep-tissue background signal. Clinical evidence suggests that NBI is an effective tool for predicting lesion histology and defining margin [6, 7]. However, it provides little to no improvement in lesion detection rates [8, 9]. Other technologies have been explored to leverage cellular-level structural contrast between normal and adenomatous tissues, including elastic scattering spectroscopy [10], confocal endomicroscopy [11], and optical coherence tomography [12, 13]. Although these modalities are promising for the lesion classification and margin delineation, their minuscule field-of-view (FoV) inhibits the surveillance of large areas of the gastrointestinal tract, making them impractical for lesion detection. In addition to endogenous sources of contrast, exogenous fluorescent dyes have been designed to selectively bind to tumor-associated molecules for the detection and delineation of lesion [14, 15]. The specificity, cost, and challenges associated with dye administration remain significant obstacles to clinical adoption.

Both technical challenges associated with imaging through flexible endoscopes, as well as safety barriers associated with human testing, have hindered the *in vivo* exploration of novel optical enhancement modalities in gastroenterology. To circumvent these challenges, researchers often characterize resected, *ex vivo* tissue as a surrogate for *in vivo* tissue. Unfortunately, resected tissues undergo rapid chemical and morphological changes, introducing significant error to the measured biomarkers [16, 17]. To enable *in vivo* imaging, researchers have developed probes and imaging bundles that can be inserted through the endoscope instrument channel [18, 19, 20]. A major technical challenge of this approach is the limited spatial resolution of the imaging bundles, which reduces the image resolution by several orders of magnitude compared to conventional flexible endoscopes. Moreover, the utilization of the instrument channel for imaging requires frequent insertion and removal of the probe, significantly lengthening procedures. Another approach is to modify the clinical illumination source to facilitate the transmission of light through the endoscope’s fiber optic light guide [21]. Although this approach significantly improves image resolution, it also eliminates the ability to image with clinical illumination, preventing the delivery of the clinical standard-of-care. To improve cancer detection in endoscopy, enhanced imaging techniques must be capable of non-contact visualization of a wide field-of-view at varying working distances, with HD resolution, and at a video rate.

Here we introduce Multi-contrast Laser Endoscopy (MLE) - a research platform for acquiring multimodal images of gastrointestinal tissue *in vivo*. This approach retrofits the internal light guides of a clinical colonoscope, incorporates a multi-contrast laser illumination source, and utilizes a custom hardware-software architecture for synchronized illumination control and real-time frame capture, processing, and display. The MLE system can display conventional standard-of-care WLE images while acquiring multimodal data in the background, facilitating its integration into the clinical workflows. We validate multispectral, perfusion, and topographical MLE modalities using calibrated imaging targets and phantom tissue models. MLE’s capabilities are then demonstrated in a first-in-human pilot study of participants undergoing standard-of-care colonoscopy. Our results demonstrate that MLE provides real-time, high-definition, wide field-of-view contrast enhancement of pathological gastrointestinal tissues *in vivo*.

## Results

### Design of a clinically translatable MLE system

**Figure 1:**
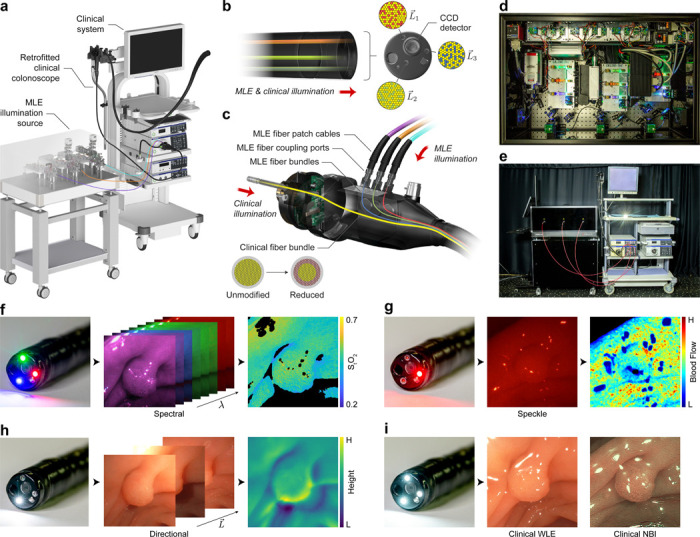
The Multi-contrast Laser Endoscopy (MLE) system for in vivo gastrointestinal imaging. **a** MLE is built around a retrofitted clinical colonoscope that interfaces with a standard clinical endoscopic imaging system while accepting multi-contrast laser illumination. **b, c** The internal light guide of a clinical colonoscope was replaced with a custom fiber optic bundle for transmitting both clinical and MLE illumination. MLE illumination is coupled through three fiber ports installed in the connector-end of the scope (c). Both the clinical fiber bundle and MLE fiber bundles map to three point sources surrounding the CCD detector at the distal tip of the scope (b). **d** Photograph of the multi-contrast illumination source constructed from lasers with varying wavelengths and coherence lengths. **e** Photograph of the retrofit clinical colonoscope, clinical endoscopic imaging system, and multi-contrast laser illumination source. **f - i** Tubular adenoma (5 mm diameter) in the descending colon imaged during a screening colonoscopy. **f** Multispectral frames were acquired with illumination from 8 narrow-band wavelengths (λ) and used to estimate total oxygen saturation. **g** High-coherence illumination generates laser speckle contrast for visualizing local tissue perfusion. **h** Point sources surrounding the detector $\left(\vec{L}\right)$ were independently toggled to acquire directionally illuminated images to enhance tissue surface shape. **i** The system retains full standard-of-care clinical imaging capabilities for acquiring white light endoscopy (WLE) and narrow band imaging (NBI) illuminated frames.

To enable high-throughput experimental imaging of gastrointestinal tissues in the clinical setting, we retrofitted a clinical colonoscope to accept a custom-built multi-laser illumination source and interface with standard clinical endoscopic imaging systems (Fig. 1a). Unlike previous systems that use imaging fiber bundles for data acquisition, our system leverages the optimized optics and CCD sensor within the endoscope to acquire HD (1080 × 1350 pixels), video rate (29.97 interlaced frames per second), wide FoV (170°), large depth-of-field (DoF; 5–100 mm) image data. In addition, the retrofitted colonoscope design enables rapid toggling (<1 s) between clinical and MLE illumination modes, minimizing disruption to the clinical workflow.

To facilitate data acquisition with both clinical WLE and MLE illumination modes, a colonoscope was internally modified with a custom fiber optic light guide (Fig. 1b-c). The light guide includes three MLE fiber bundles that are accessible for optical coupling through three ports added to the connector-end of the colonoscope (Fig. 1c). Fibers from the MLE bundles are randomly mixed with fibers from the clinical illumination bundle at the distal end of the colonoscope (Fig. 1b), allowing tissue to be imaged with light from either illumination source. This approach reduces the barrier to clinical translation, as the system maintains full clinical functionality for delivering the standard-of-care, and modifications are limited to non-patient contacting surfaces of the colonoscope, minimizing any added risk of infection or other complications.

The multi-laser illumination source was constructed using 15 laser diodes with 8 narrowband wavelengths (406–657 nm) and varying coherence lengths to acquire speckle and speckle-free images (Fig. 1d). A light modulation controller was developed to synchronize the pulse widths of the laser diodes with the frame capture rate of the colonoscope CCD sensor, and a custom software platform was developed to acquire, display, and store raw uncompressed frames from the clinical endoscopy system in real time. The system includes an auto-exposure function to prevent over- and under-exposed frames during the frequent changes in working distance typical in endoscopy. The illumination source was built on an optical breadboard enclosed in a cart for transport to and from the clinic (Fig. 1e). The MLE system can rapidly acquire multiple contrast enhancement channels, including spectral, flow, and topographic measurements, in addition to clinical WLE and NBI (Fig. 1f-i).

### Widefield measurement of spectral reflectance and oxygen saturation

**Figure 2:**
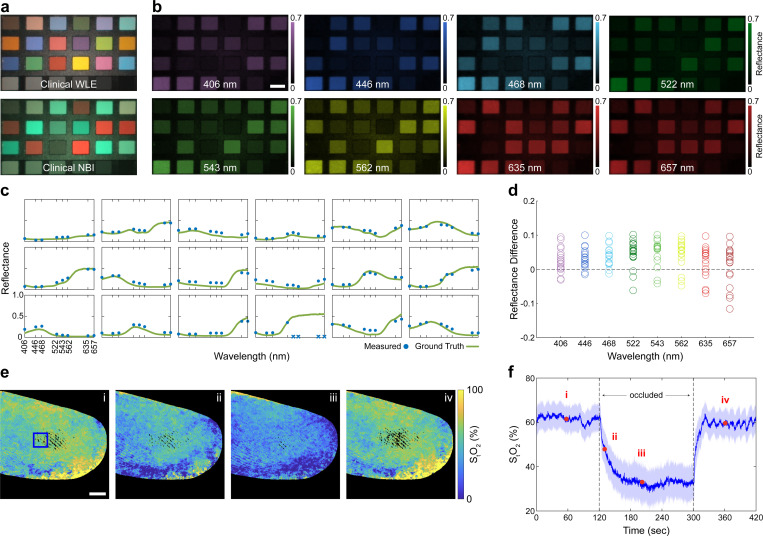
MLE enables widefield spectral imaging for interrogating molecular chromophore changes in tissue. **a** Images of a Macbeth color target acquired with clinical WLE and NBI illumination. **b** Multispectral reflectance images of the color target acquired with MLE. Reflectance measurements were normalized using images of a white reference target. **c** Mean spectral reflectance values from the first three rows of patches in (b) and ground truth spectra measured with a spectrometer. Four measurements with saturated pixels are indicated by an X. **d** Relative difference between MLE reflectance and reference spectrometer measurements for each wavelength channel. **e** Total oxygen saturation $\left({\text{S}}_{\text{t}}{\text{O}}_{2}\right)$ measurements of a human index finger before (i), during (ii,iii), and after occlusion (iv) by a pressure cuff. Black regions indicate saturated, under-exposed, and background pixels. **f** Time series ${\text{S}}_{\text{t}}{\text{O}}_{2}$ values from the region indicated by the blue square in (e). Solid line and shaded area in (f) indicate the ${\text{S}}_{\text{t}}{\text{O}}_{2}$ mean and standard deviation, respectively. See Supplementary Video 1 for measurements over the complete time course. Scale bars = 4 mm

Diffuse reflectance spectroscopy has been widely studied as a non-contact, label-free method for revealing differences in underlying tissue makeup and structure. To evaluate the accuracy of multispectral reflectance measurements acquired with MLE, we imaged a color calibration chart with 18 patches that exhibit distinct spectral reflectance signatures. Compared to clinical WLE and NBI, which capture two to three bands of spectral information (Fig. 2a), MLE measures spectral reflectance at 8 distinct wavelengths (Fig. 2b). Qualitative inspection of the spectral reflectance values measured by MLE and a reference spectrometer shows strong agreement for each Macbeth patch (Fig. 2c). Quantitative comparison with the reference spectra results in a mean absolute reflectance error of 0.05 ± 0.01, with each spectral channel error centered near 0 (Fig. 2d).

Spectral information can be used to map absorbance and quantify hemoglobin concentration and total oxygen saturation $\left({\text{S}}_{\text{t}}{\text{O}}_{2}\right)$ levels, both of which have been observed to change with cancer progression [22]. To assess the ability of MLE to detect changes in these biomarkers, we conducted a finger occlusion trial where tissue ischemia was systematically induced by inflating a pressure cuff. The tip of the colonoscope was placed above the index finger, while reflectance measurements were acquired cyclically at a rate of 7.5 full spectral measurements per second. The reflectance images were converted to absorption (see Methods), and the relative concentrations of oxy- and deoxy-hemoglobin were estimated by fitting the absorption data to known reference spectra. Sample ${\text{S}}_{\text{t}}{\text{O}}_{2}$ maps from before (i), during (ii, iii), and after (iv) occlusion are shown in (Fig. 2e). ${\text{S}}_{\text{t}}{\text{O}}_{2}$ values from a 50 × 50 pixel region of interest during the full-time course are shown in Fig. 2f. The measured oxygen saturation dropped from a baseline of 61.5 ± 5.9% (i) to 33.0 ± 8.0% during occlusion (iii), before returning to 59.6 ± 6.0% (iv) after releasing the pressure cuff. The oxygen saturation measurements acquired with MLE are consistent with measurements acquired with a benchtop imaging system [23] and demonstrate the sensitivity of MLE to measuring functional differences in tissue.

### Laser speckle contrast enhancement of flow regions

**Figure 3:**
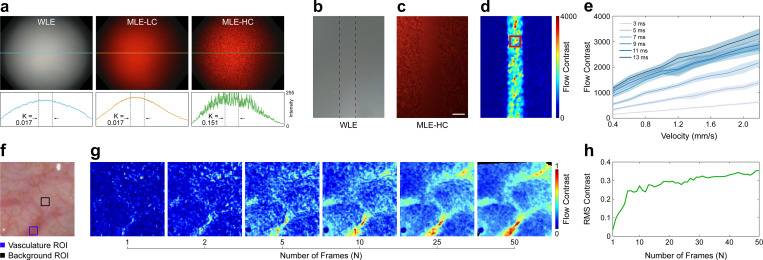
MLE is sensitive to differences in blood flow. **a** Images of a uniform scattering medium under WLE, low coherence laser (MLE-LC), and high coherence laser (MLE-HC) illumination. Displayed below are line profiles from the red color channel of each image, along with speckle contrast (K) values taken from ±100 pixels around the center of the full-width half maximum of each profile. Low-coherence laser illumination provides similar illumination uniformity and smoothness to WLE. **b, c** Images of a microfluidic phantom under WLE (b) and MLE-HC (c) illumination. A polystyrene bead suspension was pumped through a microfluidic phantom channel (dashed lines in b) at a velocity of 1.1 mm/s to simulate blood flow. **d** Flow contrast map generated by applying laser speckle contrast analysis to (b). A 9 × 9 median filter was applied for visualization. **e** Flow contrast values from the region indicated by the red square in (d) across a range of flow velocities and laser pulse widths. Solid lines and shaded areas indicate the flow contrast mean and standard deviation from 100 flow contrast images, respectively. **f** WLE image of arterioles and capillaries in the soft palate. **g** Flow contrast maps of the field of view (FoV) shown in (f) with an increasing number of registered and averaged flow contrast frames. See Supplementary Video 2 for a flow contrast video sequence. Black regions indicate either saturated or underexposed pixels, or pixels outside the FoV due to motion registration. **h** RMS contrast for flow contrast frames between the regions indicated in (f). All values are reported in arbitrary units. Scale bar = 100 μm

In addition to the low-coherence laser diodes (MLE-LC) used for spectral imaging, a high-coherence laser (MLE-HC) was integrated into the multi-contrast illumination source to produce well-defined speckle for laser speckle contrast imaging (LSCI). As shown in Fig 3a, MLE-LC illumination produce images with speckle contrast (K) similar to clinical WLE, while MLE-HC illumination produces images with an order of magnitude greater speckle contrast.

To characterize the ability of MLE to quantify flow with LSCI, a microfluidic flow phantom was fabricated from polydimethylsiloxane (PDMS) doped with titanium dioxide (TiO_2_) and India ink to simulate optical scattering and absorption by tissue. An open 500 × 150 μm channel was incorporated 100 μm below the phantom surface. A suspension of polystyrene beads was mixed to emulate the optical scattering of human blood, and a syringe pump was used to pump the microsphere solution through the channel at velocities spanning flow rates typically observed in microvasculature (0.4 mm/s - 2.2 mm/s). Fig. 3b,c show images of the phantom acquired with clinical WLE and MLE-HC illumination. In both images, the flow chamber is indistinguishable from the static surface surrounding it. However, after processing the MLE-HC image with LSCI, which spatially analyzes the reduction in speckle intensity caused by motion, the flow region becomes visually identifiable (Fig. 3d). In addition to improving the visual contrast of the flow regions, LSCI can quantify differences in flow velocity (Fig 3e). The results are consistent with previous studies acquired with non-endoscopic systems, in which the flow contrast increases with flow velocity and exposure time [24, 25].

To assess the sensitivity of MLE to variations in blood flow in the presence of motion *in vivo*, we imaged arterioles and capillaries in the human soft palate mucosa *in-vivo* (Fig 3f). The flow contrast was improved by registering sequential speckle contrast frames, via interlaced color imaging, and averaging them (see Methods). Qualitative inspection of the flow contrast maps shows that the vasculature becomes distinguishable from the background tissue with an average of 10 or more frames (Fig 3g). To quantify this improvement, the root-mean-square (RMS) contrast [26] between the vasculature and background regions of interest (ROI) was calculated for each temporal window size (Fig 3h). These results demonstrate that RMS contrast increases significantly over the first 0–7 frames, then gradually improves as the number of frames increases.

### Surface shape enhancement with photometric stereo

Tissue shape is an important cue for detecting and classifying adenomas [27]. Modern colonoscopes illuminate the field through multiple light sources oriented around the camera. While this approach minimizes shadowing and improves illumination quality, it also minimizes topographical shading variations that signal surface shape. To enable direct measurement of colon topography, we captured directionally illuminated images by sequentially toggling laser sources that map to the three point sources at the tip of the scope $\left({\vec{L}}_{1}-{\vec{L}}_{3}\right)$. This sequence of images was used to computationally reconstruct surface topography using photometric stereo endoscopy (PSE) [28].

**Figure 4:**
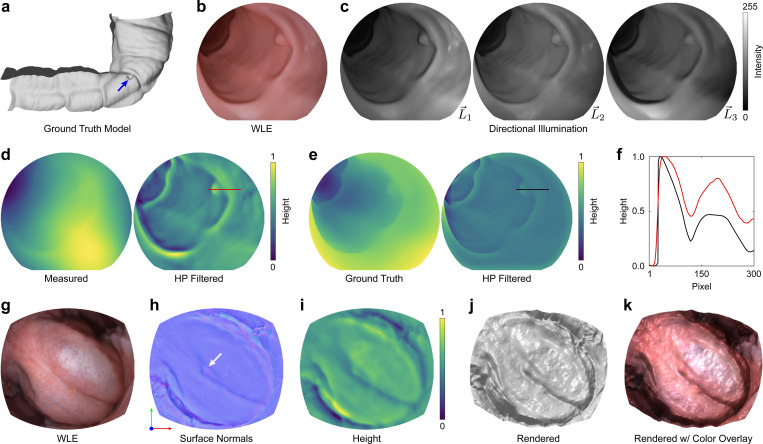
MLE enhances visual contrast of subtle changes in mucosal surface. **a** Virtual 3D colon model with a 7 mm diameter tubular adenoma (blue arrow). **b** WLE image of a silicone cast of the model. **c** MLE images of the phantom acquired with directional illumination from the three point sources $\left(\vec{L}\right)$ at the tip of the colonoscope (${\vec{L}}_{1}$, upper right; ${\vec{L}}_{2}$, upper left; ${\vec{L}}_{3}$, lower left). **d** Surface height maps estimated from the images in (c) before (left) and after (right) applying a high-pass (HP) spatial frequency filter. **e** Ground truth surface height maps rendered from the virtual 3D colon model before (left) and after (right) applying a HP spatial frequency filter. **f** Comparison of the normalized surface height values from the red (measured) and black (ground truth) lines shown in (d) and (e). **g** WLE image of the ventral surface of the tongue. **h,i** HP filtered surface normal and height maps computed from MLE directionally illuminated images. The white arrow points to a diminutive surface feature that is not visible in the WLE image. **j,k** Surface height map from (i) rendered using a virtual directional illumination source, a Phong reflection model, with a constant albedo (j) and a color overlay (k). Surface normal maps are displayed with the X/Y/Z components stored in separate R/G/B color channels. The components are linearly scaled from ±1 to 0–255. Height values are normalized. See Supplementary Video 3 for ventral tongue measurements over the complete time course.

We characterized the accuracy of PSE topography estimation by measuring a silicone colon phantom and comparing surface reconstructions to the known model profile (Fig. 4a). We captured images of the phantom with both WLE and directional illumination (Fig. 4b,c). We then estimated the surface normal vector of each image pixel using a fixed geometry between the camera and the light sources. This surface map was integrated to produce a surface height map (Fig. 4d). Low-spatial frequency error in the height map resulting from the unknown working distance between the endoscope and the tissue surface was attenuated by applying a high-pass filter. The high-spatial frequencies of the measured height map show strong agreement with the ground truth (Fig. 4e), as shown in Fig. 4f, achieving a mean absolute error of 0.11.

PSE was then tested in human tissue by free hand imaging the ventral tongue (Fig. 4g). The directionally illuminated images were registered and spatially aligned before estimating high-spatial frequency surface normal and height maps (Fig. 4h,i), which improves the visual contrast of diminutive topographical features that were not visible in WLE (Fig. 4g). The height map was also rendered for qualitative visualization with and without the WLE measurement overlay (Fig. 4j,k).

### Application of MLE during colonoscopies

We conducted a clinical study with MLE, composed of 20 participants scheduled for colonoscopy at the Johns Hopkins Hospital. We imaged 31 polyps from these patients with both WLE clinical and MLE research illumination modes (Supplementary Table 3). Only precancerous polyps (tubular, tubulovillous, or serrated histology) confirmed with histopathology of the resected samples were included in the study.

**Figure 5:**
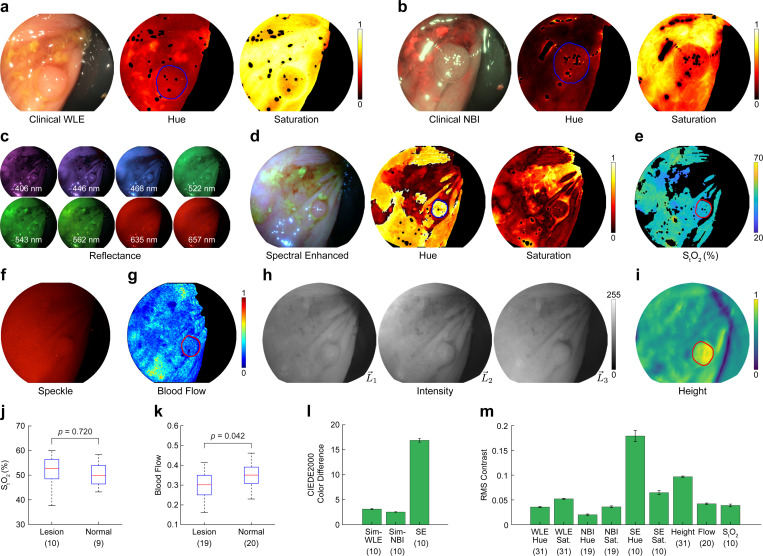
MLE enhances adenoma contrast during screening colonoscopies. **a, b** Tubular adenoma (4 mm diameter) in the ascending colon visualized with clinical white light endoscopy (WLE) and narrow band imaging (NBI). The WLE and NBI frames were converted to normalized hue and saturation for brightness-independent analysis of color contrast. **c** Multispectral reflectance images acquired at eight narrowband wavelengths with MLE. **d** Spectral enhanced image with normalized hue and saturation channels simulated using the reflectance data in (c). **e** Spatially-mapped total oxygen saturation recovered from the reflectance data in (c). **f, g** High-coherence (HC) laser speckle image and spatially-mapped blood flow (normalized). **h, i** Directionally illuminated images and recovered height map (normalized). **j, k** Average ${\text{S}}_{\text{t}}{\text{O}}_{2}$ and blood flow (k) values from lesion and normal tissue regions in each sample. The red line shows the median, blue box the interquartile range, and black whiskers the non-outlier range (n=1 outlier removed from normal ${\text{S}}_{\text{t}}{\text{O}}_{2}$ and lesion blood flow). P values are from two-sided Mann–Whitney U tests. **l** CIEDE2000 color difference computed between pixels from normal and lesion tissue regions in each sample (data are mean ± s.e.m.). **m** RMS contrast values for each modality computed between normal and lesion tissue regions (data are mean ± s.e.m.). The number of samples analyzed is listed below each modality in parenthesis, and an outline of samples is provided in Supplementary Table 3. Red and blue lines in (a)-(i)) denote the boundary between normal and lesion tissue used for analysis. Black regions indicate saturated, under-exposed, and pixels beyond the field of view due to image transformation following registration. See Supplementary Videos 4–6 for videos of measurements over the complete time course.

An example lesion from the dataset (4 mm tubular adenoma, ascending colon) is shown in Fig. 5. WLE visualization exhibits poor contrast between the adenomatous tissue and normal tissue. Both regions appear yellow with subtle perceivable differences in texture or surface shape (Fig. 5a). Similarly, under NBI illumination, the color contrast is poor, as both types of tissue have a blue tint (Fig. 5b). However, NBI offers some improvement in visualizing the disrupted surface texture compared to WLE, likely because of the shallow penetration depth of the illumination wavelengths. A lack of color contrast is also evident in the hue and saturation channels of both WLE and NBI images.

Using spectral reflectance data acquired with MLE (Fig. 5c), we simulated color images while varying the camera’s spectral transmission curves to optimize the color contrast between normal and adenomatous tissues (Supplementary Fig. 7). The color contrast in the spectral enhanced (SE) image was significantly improved compared to clinical WLE and NBI, with the lesion appearing darker and red relative to the surrounding tissue (Fig. 5d). The improvement in color contrast can also be seen in the hue and saturation channels. Quantitatively, spectral enhanced images from the dataset exhibited a five-fold increase in visually perceptible color difference (Fig. 5l) and a three-fold increase in RMS contrast compared to WLE and NBI (Fig. 5m).

We also explored using functional measurements as a source of image contrast. The reflectance images from MLE were used to spatially map total oxygen saturation (Fig. 5e). The average total oxygen saturation $\left(\bar{x}=51.3\text{\%}\right)$ is consistent with prior work [29], but the differences between polyp and normal tissue were not statistically significant (Fig. 5j). Laser speckle illuminated frames (Fig. 5f) were acquired to spatially map blood flow with LSCI (Fig. 5g). The mean blood flow measured with LSCI was 9.0% less in polyp tissue than in normal tissue, and the difference in average blood flow for polyp and normal regions was found to be statistically significant (Fig. 5k). In some samples, the decrease in blood flow was relatively uniform within the margins of the polyps, making them visually identifiable, whereas in other cases, the blood flow was non-uniform in appearance. The poor distinction between tissue types is reflected in the quantitative analysis, where the modalities have similar RMS contrast values as WLE and NBI (Fig. 5n).

In contrast to WLE and NBI, height reconstruction from MLE directional illumination (Fig. 5h) revealed a distinct change in surface topography at the lesion margins (Fig. 5i), offering a valuable signal for lesion detection and segmentation. The RMS contrast provided by surface shape resulted in an approximately two-fold improvement relative to WLE and NBI.

## Discussion

While colonoscopy has long been considered the gold standard for preventing and detecting CRC, recent evidence suggests that its efficacy in reducing CRC mortality may be less robust than initially believed. This efficacy is limited in part from missed lesions that arise from reliance on color and texture information to differentiate diseased and healthy tissue. To improve lesion contrast contrast, optical image enhancement modalities are being developed to offer label-free, real-time information that complements WLE. Here, we introduced an MLE platform to investigate new sources of optical contrast during *in vivo* gastrointestinal imaging. Unlike other techniques that use custom scopes or probes, this approach integrates directly with the clinical imaging system and leverages the optimized internal optics and image sensor. The result is a system with an standard-of-care spatial resolution (1.5M pixels), FoV (170°), and DoF (5–100 mm). Importantly, the system can quickly toggle between clinical and research illumination modes, allowing its practical use in large clinical studies.

The methods and results presented here come with several important limitations. First, regarding the MLE system, pixel values captured by the colonoscope’s CCD sensor are subject to non-linear and black-box transformations (e.g. gamma correction) by the clinical video processor. Although benchtop characterization with a color target demonstrated robust measurement accuracy, further performance gains could likely be achieved by inverting or circumventing these transformations. Additionally, frames are sequentially acquired with different illumination parameters, and spatially aligned using image registration during post-processing. The frequent movement of the scope and surrounding tissue limits the number of frames, and consequently, the number of illumination channels that can be reliably registered. It is also important to note that, although our results demonstrate that MLE improves the RMS contrast between lesions and surrounding tissue, our study design did not test if this approach translates into an improvement in lesion detection rates. The impact on lesion detection merits further investigation, possibly through a randomized controlled trial that compares the adenoma detection rate (ADR) between WLE and MLE groups.

With simple modifications, the MLE research platform can be utilized to address several key opportunities in endoscopy screening. First, the laser diodes within the illumination source can be interchanged to evaluate different wavelengths for lesion endogenous or exogenous contrast. These wavelengths could be extended to the ultraviolet range for autofluorescence imaging or to the near-infrared range for evaluating deep and background-free fluorescence imaging with molecularly-targeted dyes [15]. Furthermore, the high-coherence channel can be used in conjunction with speckle-illumination spatial frequency domain imaging to quantify bulk tissue optical properties and tune the imaging depth [30, 31]. Second, the feature-rich information acquired by MLE could improve the performance of deep learning-based detection, classification, and margin delineation. Third, a learned-sensing approach enables the joint optimization of illumination parameters (wavelength, coherence, direction) with the neural network’s filter weights, thereby optimizing the measurement system for machine interpretation [32]. The learned illumination weightings could then be used to acquire computational images in a single snapshot, eliminating the need to register frames. Further, the computational image could then be digitally transformed to a WLE image for interpretation by the clinician [33]. Learning-based methods require large volumes of data, and MLE addresses this challenge with its relative ease of obtaining large quantities of *in vivo*. Finally, the system could be used to improve esophageal or gastric cancer screening, or for applications beyond lesion detection, such as the quantitative grading of fibrosis in inflammatory bowel disease or the detection of Barrett’s esophagus.

In conclusion, we introduced a multi-contrast laser endoscopy platform for obtaining *in vivo* clinical data through a flexible endoscope. This approach overcomes many of the safety and regulatory hurdles associated with human testing while leveraging the decades of image sensor and micro-optics developments incorporated into a clinical endoscope. We showed lesion contrast enhancement using multispectral, perfusion, and topographic imaging, demonstrating the potential of this platform to explore a variety of biophotonics modalities in large-scale clinical studies.

## Methods

### Retrofitted clinical colonoscope design

The MLE system is constructed around an adult video colonoscope (CF-HQ190L; Olympus America Inc., Center Valley, PA, US) modified to enable illumination with clinical and research illumination sources (Fig. 1). The colonoscope’s internal fiber optic light guide was replaced with a custom fiber optic bundle that includes multiple MLE fiber bundles that allow external illumination. The custom bundle was constructed from 35 μm, 0.55 NA borosilicate fibers with custom-machined ferrules compatible with the internal components of the scope (Gulf Fiberoptics, Oldsmar, FL, US).

To make the internal MLE fiber bundles accessible for optical coupling, the fiber bundle ferrules were mounted within stainless steel SMA fiber ports added to the connector-end of the colonoscope. The fiber port additions and custom fiber bundle installation were made by Fibertech Medical (Timonium, MD, US). See Supplementary Section 1 and Supplementary Fig. 1 for additional fabrication details.

### Multi-contrast illumination source

The multi-contrast illumination source included three RGB laser units to emulate standard white light (enabling all three RGB units) and directional (enabling a single RGB unit) illumination. The RGB units were constructed from 446 nm, 522 nm, and 635 nm multimode laser diodes with collimation and combining optics. To increase the diversity of wavelengths available for multispectral imaging, five additional multimode laser diodes with wavelengths centered at 406 nm, 468 nm, 543 nm, 562 nm, and 657 nm were included in the system. These wavelengths were selected to coincide with points at which the absolute values of the ratios of oxy- and deoxy-hemoglobin are either maximal or close to one (isosbestic) to facilitate tissue oxygen saturation mapping. A 639 nm single longitudinal mode laser (coherence length > 40 m) was included to illuminate with laser speckle for flow contrast imaging. The laser diodes were powered by drivers equipped with transistor–transistor logic switching for pulse width modulation. The drive current for each laser diode was tuned so that the total output power emitted by the scope with all diode channels enabled was less than the maximum output power emitted by an unmodified colonoscope and clinical light source (330 mW). Laser diode temperature was regulated by PID-controlled thermoelectric cooler drivers. Laser diode output power was continually measured using photodiode monitoring units. See Supplementary Section 2, Supplementary Figs. 2 and 3, and Supplementary Tables 1 and 2 for optical components and layout.

### Light modulation controller

Laser diodes in the multi-contrast illumination source were triggered using a custom light modulation controller printed circuit board (PCB). The PCB contained a video-sync separator chip (LM1881; Texas Instruments, Dallas, TX, US) for synchronizing the light triggering with the colonoscope frame acquisition rate. The PCB also included a microcontroller unit for controlling the output power of each diode with pulse width modulation. See Supplementary Section 3 and Supplementary Fig. 4 for additional design details and a circuit schematic.

### System architecture

The clinical endoscopic imaging system consists of a video processor (CV-190; Olympus America Inc., Center Valley, PA, US), xenon light source (CLV-190; Olympus America Inc., Center Valley, PA, US), and two display monitors. The connector end of the colonoscope plugs into the clinical light source and video processor for optical coupling with the arc lamp and transmission of the CCD signal to the video processor. Control of the MLE system was managed by a workstation (Dell Precision 5820T, Intel i7–9800X 3.80 GHz 8-core Central Processing Unit (CPU); 64.0 Gb physical memory) running Microsoft Windows 10. An HD frame grabber (Orion HD, Matrox Imaging, Montreal, Canada) captured and stored uncompressed video from the clinical video processor. A custom C++ application was developed to enable real-time data acquisition, control, and display of the MLE system. The application included threads for (1) a command line interface to receive user inputs for transitioning between MLE illumination modes, (2) asynchronously processing data received by the frame grabber and updating the displays, and (3) communicating with the light modulation controller. Multiple buffering was employed to enable concurrent frame capturing and processing while also minimizing the risk of dropped frames. Image processing was offloaded to an Nvidia TITAN Xp graphics processing unit installed within the workstation. To maintain a well-exposed image with changes in working distance and tissue reflectance, pulse width updates were continuously sent to the light modulation controller. Updated pulse widths were computed on the host desktop using an auto-exposure technique based on an adaptation of the secant root-solving method [34]. A light pulse was flashed and detected in the acquired frames to synchronize the MLE system with the frame acquisition delay of the clinical system. See Supplementary Section 4 and Supplementary Fig. 5 for a detailed description of the hardware and software components and interfaces.

The MLE system was pre-programmed with white light, topographic, laser speckle, and multispectral illumination modes. Each mode was defined with a laser diode sequence, relative diode intensities, color channels used for auto-exposure, and post-processing steps for research and clinical video display outputs. A summary of the pre-programmed illumination modes is shown in Supplementary Fig. 6.

### Image processing

Raw video sequences acquired by the MLE system were post-processed with custom scripts executed in Matlab 2024b (The MathWorks, Inc., Portola Valley, CA, USA). A dark frame was subtracted from the frames to remove the contribution of dark current. Deinterlacing was performed by splitting odd and even pixel rows into separate images and resizing them with bilinear interpolation to the original image height. Lens distortion was removed using intrinsic parameters measured with a checkerboard target [35].

Spectral illumination frames were split into substacks each containing 8 image fields (one complete spectral cycle). Electronic and residual speckle noise was reduced by spatially averaging neighboring pixels with a 5 × 5 Gaussian filter (σ=0.5). The image intensity values were transformed to reflectance by correcting for the system response (diode output power, Bayer transmissivity, sensor quantum efficiency) and duty cycle (diode pulse width length). The system response for each diode was measured by imaging a reference target and computing the mean intensity of each spectral channel. To correct for movement of the tissue and colonoscope between acquisitions, each wavelength channel was registered to the 561 nm image using a mutual information criterion [36], and the resulting affine transforms were applied to spatially align the frames.

Total oxygen saturation at each (u,v) pixel was estimated from multispectral absorbance (A) calculated from reflectance measurements (R) at each wavelength (λ) using the modified Beer-Lambert law:

(1) $$A(u,v,\lambda )=-\log_{10}(R(u,v,\lambda ))\;\;=\left[c_{\text{Hb}{\text{O}}_{\text{2}}}(u,v)\cdot \epsilon_{\text{Hb}{\text{O}}_{\text{2}}}(\lambda )+c_{\text{Hb}}(u,v)\cdot \epsilon_{\text{Hb}}(\lambda )\right]\cdot L+O$$

where $\epsilon_{\text{Hb}{\text{O}}_{\text{2}}}$ and $\epsilon_{\text{Hb}}$ are measured molar extinction coefficients of oxy- and deoxy-hemoglobin [37], $c_{\text{Hb}{\text{O}}_{\text{2}}}$ and $c_{\text{Hb}}$ are the concentrations of HbO_2_ and Hb in the sample, L is the optical pathlength, and O is an additional term that accounts for attenuation by other chromophores and optical scattering [38, 39]. To simplify this expression, we assumed a constant optical path length across wavelengths, allowing us to recover $c_{\text{Hb}{\text{O}}_{\text{2}}}$, $c_{\text{Hb}}$, and LS from the measured reflectance spectra using linear non-negative least squares regression. Total oxygen saturation was then computed as the relative concentration of oxygenation hemoglobin $\left(c_{\text{Hb}{\text{O}}_{\text{2}}}\right)$ to total hemoglobin $\left(c_{\text{Hb}{\text{O}}_{\text{2}}}+c_{\text{Hb}}\right)$. Reflectance values were kept within the range (0,1) by dividing by the maximum reflectance value within the multispectral data cube plus a small constant. For the finger occlusion data presented in Fig. 2, reflectance values were normalized with respect to the 659 nm channel [40] and converted to absorption using the Kubelka-Munk model [41] with scattering explicitly modeled using parameters from [42], and melanin was included in the unmixing using absorption values provided in [43].

Color images were simulated using the spectral reflectance measurements, the transmission spectrum of a Bayer camera filter [44], and the measured output illumination spectra from the clinical light source. Optimized weights for the simulated spectrally enhanced images were identified using gradient descent optimization, with an objective function designed to maximize the CIEDE2000 color difference $\left(E\delta_{00}\right)$ [45] between 100 pixels sampled from normal and polyp tissue regions in each image. WLE and NBI images were also simulated, allowing the CIEDE2000 color difference to be computed for the same pixels as the spectral enhanced images. Prior to processing, the reflectance channels for each wavelength were rescaled to the average intensity for that wavelength across the dataset. Each pixel was normalized so that its maximum color channel value equaled 0.8 to remove the effect of brightness in the color difference calculation [46]. For visualization, the images were scaled so that the mean intensity of the image was equal to 0.4. See Supplementary Fig. 7) for additional implementation details.

Laser speckle-illuminated frames were post-processed to map perfusion. The MLE-HC and RGB lasers were alternately illuminated to interlace color and speckle contrast images in each full acquired frame. These frames were deinterlaced into two image stacks: laser speckle-illuminated frames originating from odd fields (n=1,3,…,N−1) and white light-illuminated frames originating from even fields (n=2,4,…,N). Next, the laser speckle-illuminated frames were converted to laser speckle contrast (K) frames using:

(2) $$K=\frac{\sigma }{\langle I\rangle },$$

Here, σ and 〈I〉 are the standard deviation and mean within a 5 × 5 spatial window centered at each pixel [47]. Speckle contrast frames were converted to flow contrast frames (V) using $V=\frac{1}{K^{2}}$ [48]. To improve the blood flow signal, the blood flow frames were averaged within a rolling temporal window of 15 frames. Before averaging, blood flow frames within the window were spatially aligned using registered features from the corresponding white light illuminated frames. The white light illuminated frames were registered by minimizing the mean square error between images with gradient descent optimization.

Surface topography was recovered from directionally illuminated images using photometric stereo [49]. For simplicity, measured intensity values from the directionally illuminated frames were related by assuming a diffuse Lambertian reflectance model

(3) $$I_{n}=\frac{\rho_{d}}{\pi }\cdot {\hat{s}}_{n}\cdot \hat{n},$$

where I is the measured intensity, $\rho_{d}$ is a scalar term denoting the surface albedo, $\hat{s}$ is the pre-calibrated directional vector of light source n, and $\hat{n}$ is the surface normal to be estimated. The observed intensities and corresponding light source directions were combined with Equation 3 to formulate a linear system of equations for each pixel:

(4) $$\underset{\text{N}\times 1}{I}=\underset{\text{N}\times 3}{\hat{s}}\times \underset{3\times 1}{\vec{n}}.$$

Here, $\vec{n}$ is a non-unitary vector with magnitude $\frac{\rho_{d}}{\pi }$ and direction $\hat{n}$, and N is the number of light sources. Surface normals were estimated for every pixel within a rolling window of three image fields acquired with unique point sources. Before solving for the normals, specular reflections were inpainted, and the directional images were registered and spatially aligned by minimizing the mean square error between images with gradient descent optimization. The short working distance, wide FoV, spatially-varying anisotropic light sources located near the camera, and unknown working distance in our application introduce low-spatial frequency error to the recovered surface normals. This error was removed using a high-pass filter (σ=150), which was applied by convolving the surface normal map with a low spatial frequency filter and subtracting the result from the unfiltered surface normal map, leaving only the high-spatial frequency components [28]. To reconstruct a height map, the filtered surface normals were projected to gradient space and integrated using a multigrid solver for the Poisson equation [50, 51].

### Spectral fidelity evaluation

The spectral fidelity of MLE was characterized by imaging a Macbeth Color Chart (ColorChecker Classic Nano; X-Rite, Inc., US). After allowing the system to warm up for 15 min, reference measurements of a diffuse polytetrafluoroethylene (PTFE) target (PMR10P1; Thorlabs, Inc., Newton, NJ, US) were acquired and corrected using ground truth reflectance spectra provided by the manufacturer. The PTFE target was then replaced with the color target and sample measurements (I) were acquired. Spectral data cubes were constructed from the sample and reference frames, and the sample measurements were normalized with respect to the reference frames. 60 × 60 pixel regions of interest were cropped from each patch on the color chart and used to calculate the mean reflectance value at each wavelength. Ground truth reference spectra for each patch were acquired with a CCD spectrometer (CCS200; Thorlabs, Inc., Newton, NJ, US), a fiber-coupled stabilized broadband light source (SLS201L; Thorlabs, Inc., Newton, NJ, US), and a bifurcated reflection probe (RP20; Thorlabs, Inc., Newton, NJ, US).

### Finger occlusion experiment

Sensitivity to changes in total oxygen saturation was demonstrated through an occlusion trial. A blood pressure cuff (Manual Inflate Blood Pressure Kit; Walgreens, Deerfield, IL, US) was applied to the forearm of a healthy volunteer. The volunteer’s hand was depressed into a soft clay (0716623001735; Crayola, Easton, PA, US) disk and the endoscope tip was mounted above the index finger to minimize motion. A 420 sec video was recorded with multispectral illumination. After imaging 120 seconds of baseline video, the pressure cuff was inflated to 300 mmHg to occlude blood flow for 180 sec. The pressure cuff was then released and an additional 120 sec of video was recorded. The protocol for this study was approved by the Johns Hopkins Institutional Review Board (IRB00344195).

### Microfluidic flow phantom fabrication

A microfluidic flow phantom was fabricated to assess the sensitivity of MLE to differences in blood flow velocity. The phantom included 500 μm wide and 150 μm tall channels manufactured from polydimethylsiloxane (PDMS) doped with titanium dioxide (TiO_2_) and India ink for optical scattering $\left(\mu_{s}^{\prime }∼2.0\;mm^{-1},\lambda =650\;\text{nm}\right)$ and absorption $\left(\mu_{a}∼0.01\;mm^{-1},\lambda =650\;\text{nm}\right)$, respectively. The flow channels were sealed with a 100 μm thick PDMS membrane also doped with TiO_2_ and India ink using the process described in [52]. A syringe pump (98–5457; Harvard Apparatus, Cambridge, MA, US) was used to pump the polystyrene microsphere solution through the phantom to simulate blood flow. A 1.0 mL solution of deionized water and 0.943 μm diameter polystyrene microspheres (PS03N; Bangs Laboratories Inc, Fishers, IN, US) was mixed 13.86:1 to achieve a reduced scattering coefficient comparable to that of human blood ($\mu_{s}^{\prime }∼2.0\;mm^{-1},\lambda =633\;\text{nm}$) [53]. The suspension was sonicated and transferred to a 1 mL syringe.

The solution was continually mixed using a stir rod within the syringe and a rotating magnet external to the syringe to retain a homogeneous suspension throughout the experiment. The syringe was connected to the input port of the microfluidic phantom using PEEK tubing with an inner diameter of 0.020 in (1569L; IDEX Corporation, Lake Forest, IL, US). A second segment of PEEK tubing carried the microsphere solution from the phantom’s output port to a microcentrifuge tube for collection.

### Silicone adenoma phantom fabrication and imaging

The surface measurement capability of MLE was evaluated by fabricating a phantom colon model with a known three-dimensional shape [54]. A three-part mold for the model was 3D-printed using a Form 3 printer (FormLabs, Somerville, MA, US) at a resolution of 25 μm. The mold was cast with silicone (Dragon Skin^™^, Smooth-On, Inc., Macungie, PA, US), with pigments (Silc Pig^™^, Smooth-On, Inc., Macungie, PA, US) added to replicate the color of gastrointestinal tissue. Images of the phantom model, acquired using MLE, were manually registered to the ground truth model via a custom graphical user interface, which overlaid the frames with images of the model rendered by a virtual camera. Once registration was complete, ground truth depth frames were rendered for comparison with those measured by MLE.

### Soft palette and ventral tongue imaging

To test the MLE system in an imaging environment with similar properties to that of colon tissue, the oral mucosa of the ventral tongue and the soft palate were imaged in three human participants using a protocol approved by the Johns Hopkins Institutional Review Board (IRB00279890). All participants were over the age of 18 years and were capable of giving informed consent. Enrolled subjects were seated in front of a display monitor showing the live video feed from the colonoscope. Subjects were handed the colonoscope and instructed to slowly insert the scope tip into the oral cavity. The participant used the live video feed and feedback from a study team member to navigate the scope to an ideal position for data acquisition. Once positioned, approximately 10 s of video was recorded. The participant was then given a moment to rest, and the process was repeated four additional times.

### Clinical study design

A first-in-human pilot clinical study was conducted to assess the ability of MLE to enhance lesion contrast in colonoscopy. The study enrolled Johns Hopkins Health System patients who were scheduled for a screening or surveillance colonoscopy. All participants were over the age of 18 years and were capable of giving informed consent. Patients with bleeding or hemostasis disorders, patients taking anticoagulants such as Warfarin and Clopidogrel, and patients with colitis or active bleeding were excluded from the study. Informed consent was obtained in writing while the patient was in pre-procedure staging. The patient was then escorted to the procedure room and placed under conscious sedation. The MLE system was then rolled into position next to the endoscopy tower, powered on, and the optical fibers were connected to the MLE fiber ports on the retrofitted scope. The shutters on the laser source were closed, and the lasers were toggled on and allowed to warm up until research data was collected. The retrofitted colonoscope was then inserted through the anus and advanced to the cecum. As the colonoscope was withdrawn, the endoscopist examined the colon for lesions. Upon discovery of a lesion, the endoscopist alerted the study team. To avoid extending the procedure length, lesions were imaged with MLE research modalities while the resection device and associated materials were gathered and inserted through the instrument channel to the tip of the scope (typically 30 s).

Upon discovery of a lesion, the CV-190 video processor’s edge enhancement mode was toggled from *A7* to *A0* to minimize post-processing of the acquired video. The CV-190L light source was then toggled off. After sending a synchronization pulse, the MLE light source was toggled on, and data was recorded using each of the illumination modes. Once the resection device was prepared and inserted through the instrument channel, the MLE light source was toggled off, the CV-190L light source was re-enabled, and the CV-190 video processor’s edge enhancement mode was set back to *A7*. Video was then acquired in conventional WLE and NBI modes before resection. This approach yielded roughly 30 s of video per lesion. Once resected, the lesion was assigned a unique sample identifier, and ground truth histology was retrieved from the subject’s medical record post-procedure. Using this protocol, colonoscopies were performed in an identical manner as the standard of care, with the only exception being the collection of research data during the preparation of resection materials.

To reduce the risk of post-endoscopic infection arising from the use of a retrofitted colonoscope, the manufacturer of the automated endoscope reprocessors (AER) used by Johns Hopkins (Cantel Medical Corporation, NJ, USA) conducted a design review and a chemical analysis of the materials used to retrofit the colonoscope. The manufacturer approved the retrofitted scope for reprocessing in the AER (see Supplement Section 1 for AER configurations) using the standard instructions for use. All patients included in the study were longitudinally monitored post-procedure by the hospital infection control unit, and no cases of post-endoscopic infection or other adverse events were reported. Study subjects were given a single-use parking voucher as compensation for participation in the study. The study was approved as a Non-Significant Risk protocol by the Johns Hopkins Institutional Review Board (IRB00279918).

Following the procedure, frames for each illumination mode were isolated from the videos, and lesions were manually segmented by an experienced endoscopist. The frames were then post-processed and cropped so that images from each illumination exhibited a similar FoV. The location and histological label of each lesion was retrieved from the patient’s medical record.

## Supplementary Material

Supplement 1

## Data Availability

The data acquired and analyzed in this work is available online at: durr.jhu.edu/mle.
