# Supplementary material for: Multi-contrast laser endoscopy for in vivo gastrointestinal imaging
Source: ArXiv. 2025 May 15:arXiv:2505.10492v1. Preprint. [Version 1] (PMC12132275)
Supplement: Supplementary file 1 [file NIHPP2505.10492v1-supplement-1.pdf]

# Supplementary Material

## 1 Retrofitted clinical colonoscope fabrication

The internal fiber optic light guide of an adult video colonoscope was replaced with a custom fiber optic bundle. This custom bundle maps half of the fibers from the three distal sources to the clinical white light source, and the other half are mapped to three new 1 mm diameter MLE fiber bundles at the connector end of the scope. We found that the white light source was sufficiently bright to maintain standard-of-care illumination with the outer half of its illumination fibers removed (original cross-sectional area = 4.6 mm$^2$, new cross-sectional area = 2.3 mm$^2$). To make the internal MLE fiber bundles accessible for optical coupling, the fiber bundle ferrules were mounted within stainless steel fiber ports added to the colonoscope (Supplementary Fig. 1a). Three 7.4 mm diameter holes, spaced 13 mm center-to-center, were drilled through the scope connector body for the fiber ports. SMA fiber ports (ADASMA; Thorlabs, Inc., Newton, NJ, US) were installed through each hole and fastened internally using lock washers, jam nuts, and high-temperature epoxy (Cyberpoxy 5895; H.B. Fuller, Saint Paul, MN, US). Each fiber bundle was secured into a brass cylindrical adapter with an outer diameter equal to the inner diameter of the fiber port. A small set screw secured each fiber adapter within the fiber port. Crevices around each fiber port were filled with epoxy to minimize surfaces that could harbor bacteria or fluid during scope cleaning. Multimode fiber patch cables (MMF1) were used to transmit light from the illumination source, and these cables were optically butt-coupled with the MLE fiber bundles by applying light pressure between the fiber faces with custom-machined stainless steel SMA adapters (Supplementary Fig. 1b).

To reduce the risk of infection arising from the use of a retrofitted colonoscope, several precautionary measures were taken. The manufacturer of the automated endoscope reprocessors (AER) used by Johns Hopkins (Cantel Medical Corporation, NJ, USA) conducted a design review and a chemical analysis of the materials used to retrofit the colonoscope. The manufacturer approved the retrofitted scope for reprocessing in the following AER configurations using the standard instructions for use (IFU):

- ADVANTAGE PLUS and ADVANTAGE PLUS Pass-Thru AERs w/ a 2-8-611 hookup and the 1-xx-603 parameter set
- DSD EDGE and DSD-201 AERs w/ DSD-110-HU0190 and DSD-110-HU0102 hookups
- CER OPTIMA AERs w/ CLM-110-HU0108, CLM-110-HU0124, CLM-110-HU0129, and CLM-110-HU0130 hookups

## 2 Multi-contrast laser illumination source optical design

The optical layout and component list for the multi-contrast illumination source are reported in Supplementary Fig. 2 and Supplementary Table 1, respectively.

The Red-Green-Blue (RGB) laser units were constructed from 450 nm (LD-450), 525 nm (LD-525), and 637 nm (LD-637) multimode laser diodes. The laser diodes were collimated using aspheric lenses (L1), and the slow axes were magnified using cylindrical lens pairs (CLP) to attain symmetrical beam shapes. Silver (M1) and dichroic (DM1, DM2) mirrors were employed to consolidate the beams into a coaxial path. A wave rotator plate (HWP1) was used to rotate the 637 nm diode's polarization angle by 90° so that all three diodes exhibited S-polarization.

The light source also includes 405 nm (LD-405), 462 nm (LD-462), 543 nm (DPSS-543), 561 nm (DPSS-561), and 659 nm (LD-659) laser sources. Similar to the RGB units, aspheric lenses (L3, L5) and cylindrical lens pairs (CLP) were used to collimate the multimode laser diodes (LD-405, LD-462, LD-659). The 543 nm and 561 nm sources have coherence lengths long enough to generate laser speckle noise. To reduce this noise, both sources were coupled into multimode fibers (MF2), passed through a fiber oscillator (FO), and re-collimated using fixed fiber collimators (L4). Dichroic mirrors (DM3-DM6) with sharp cut-on wavelengths and a silver mirror (M2) combined the five lasers into a coaxial beam path. To improve illumination homogeneity and reduce shadows, the beam was split and coupled with RGB units 1 and 2 so that multispectral illumination was emitted from $\vec{L}_1$ and $\vec{L}_2$ simultaneously. This splitting and coupling was achieved with a 50:50 beam splitter (BS) and polarized beam splitters (PBS). All of the lasers exhibited P-polarization, causing them to be primarily reflected by the polarized beam splitters for coupling into the RGB beam paths. Any residual light not reflected by the polarized beam splitters was captured by beam blocks (BB).

A 639 nm single longitudinal mode laser (coherence length > 40 m) was combined with RGB module 3 to generate laser speckle for flow contrast imaging. To modulate the output power of the laser, the beam's polarization angle was rotated to vary the ratio of transmitted and reflected light by the polarization beam splitter. A half-wave plate (HWP2) was mounted to a direct drive rotation mount (RM) to control the beam's polarization angle programmatically.

The beams were steered using pairs of broadband dielectric mirrors (M2) into aspheric condenser lenses (L2). These lenses focused and coupled the light into 1 mm diameter multimode fiber optic patch cables (MF1). To ensure complete filling of the fibers and to minimize the risk of fiber damage, the fiber faces were positioned at approximately 1 mm before the back focal length of the aspheres. Custom-machined stainless steel SMA adapters were used to screw together the fiber patch cables and the MLE fiber ports, applying light pressure between the fiber faces for optical butt-coupling Supplementary Fig. 1b). Mechanical shutters (SHTR) were placed before the aspheres so the system could be warmed up without emitting light from the MLE illumination source.

The laser diodes were powered by drivers equipped with transistor–transistor logic (TTL) switching for pulse width modulation (PWM) exposure control. The TO-can diodes (LD-$*$) were powered by single channel constant current drivers (LPLDD-5A-24V-TP-H; Opt Lasers, Warszawa, PL) supplied by a 24 V power supply (HRP-300-24; MEANWELL Enterprises Co., Ltd., New Taipei City, TW). Laser diode temperature was regulated by PID-controlled thermoelectric cooler (TEC) drivers (TEC-20A-25V-PID-H; Opt Lasers, Warszawa, PL) powered by a 7.5 V power supply (HRP-100-7.5; MEANWELL Enterprises Co., Ltd., New Taipei City, TW). The DPSS lasers were powered by manufacturer-calibrated drivers/power supplies (PSU-H-LED/PSU-H-FDA; CNI Laser, Changchun, CN) that include dials for tuning the output power. A summary of the laser diode packages and drivers is reported in Supplementary Table 2.

The drive current for each laser diode was tuned so that the total output power emitted by the scope with all diode channels enabled was less than the maximum output power emitted by an unmodified colonoscope and clinical light source. The maximum clinical illumination power was measured by setting the clinical light source to manual illumination mode and the light output to the highest output setting. A broadband optical power meter (S470C; Thorlabs, Inc., Newton, NJ, US) was placed at the colonoscope's distal tip, and the optical output power was measured as 330 mW. Using the optical power meter, the output power of each laser diode was tuned to the values listed in Supplementary Table 2, which combined are less than the maximum clinical output power.

To correct for drift and instability in the laser output power, coverglass slips (CG) were used to pick off a small percentage ($\sim$3%) of light from each beam path for continuous monitoring. The light was measured by a photodiode (PD) mounted to a printed circuit board (PCB) fabricated by JiaLiChuang PCB (JLCPCB; Shenzhen, CN). Neutral density filters (ND) reduced the power to fall within the dynamic range of the photodiodes, and ground glass diffusers (DIFF) spread the light to reduce sensitivity to alignment. The photodiode PCB, diffuser, neutral density filter, and cover glass were combined with a 3D-printed mount and a cage adapter (CP30Q; Thorlabs, Inc., Newton, NJ, US). The electrical schematic and images of the output power monitoring unit are depicted in Supplementary Fig. 3.

All optical components were mounted to a 24 × 36 in optical breadboard (B2436F; Thorlabs, Inc., Newton, NJ, US) installed on a rolling cart (POC001; Thorlabs, Inc., Newton, NJ, US) for transport to and from the clinic. An enclosure was constructed from extruded aluminum rails and laser-cut acrylic panels to contain stray light and to protect the equipment from fluid splashes and spills. Fans were installed in the walls of the enclosure to extract heat from the enclosure. All electronics were powered by a medical-grade isolation transformer (AN120411; Toroid, Salisbury, MD, US) installed in the cart's base.

## 3 Light modulation controller

Pulse width modulation (PWM) of the laser diodes was managed by a custom light modulation controller PCB. A circuit schematic and photograph of the PCB are shown in Supplementary Fig. 4. The PCB connects to the analog separate video (S-video) output of the Olympus CV-190 video processor via a 4-pin mini-DIN cable. A low-pass filter with a cut-off frequency of 500 kHz was included to remove noise from the raw video signal. The PCB included a chip (LM1881; Texas Instruments, Dallas, TX, US) that extracts the odd/even field vertical synchronization pulses from the filtered S-video input signal. This signal was tied to a digital interrupt pin on a microcontroller unit (MCU; Teensy 4.0; PJRC, Sherwood, OR, US) to synchronize the triggering of illumination pulses with the start of each frame acquisition. Digital output pins on the MCU modulated the laser diodes. Logic level shifters (74HCT245N; Texas Instruments, Dallas, TX, US) converted the digital output pin signals from 3.3 V to 5.0 V for compatibility with the laser diode drivers. Quad timers within the MCU were used to time the pulse width lengths of the diode channels to control the exposure times. An additional quad timer was used to time the reading of the analog input voltages from the output power monitoring units. The measurements were acquired 50 µs after the rising edge of the PWM signals — a value greater than the 35 µs rise time of the photodiodes within the laser power monitoring units.

## 4 System architecture

A wireframe diagram outlining the hardware interfaces between the clinical and MLE systems is shown in Supplementary Fig. 5.

**Clinical hardware:** The clinical endoscopic imaging system consists of a video processor (CV-190; Olympus America Inc., Center Valley, PA, US), xenon light source (CLV-190; Olympus America Inc., Center Valley, PA, US), and two display monitors. The connector end of the colonoscope plugs into the clinical light source and video processor for optical coupling with the arc lamp and transmission of the CCD signal to the video processor. The colonoscope acquires images at a frequency and resolution of 29.97 interlaced frames per second and 1080 × 1350 pixels, respectively. Odd and even rows of pixels, also called image fields, are acquired by the colonoscope in an alternating fashion at a frequency twice the frame rate (59.94 fields per second). Acquired frames are transmitted to the video processor for post-processing and output via the display ports. An serial digital interface (SDI) video output from the video processor is connected to Display Monitor 1 for visualization by the clinician. The clinical system includes a second display monitor (Display Monitor 2) that is not typically utilized during screening colonoscopy procedures. This monitor was repurposed to display a blended video feed from the MLE system for easier visualization while imaging in MLE illumination modes. To reduce post-processing of the image data, the Electronic Shutter (ES) and Auto Gain Control (AGC) settings were disabled, and the Edge Enhancement mode was set to *A0* in the user settings of the CV-190 video processor.

**MLE hardware:** Control of the MLE system was managed by a Dell Precision 5820T Workstation (Intel i7-9800X 3.80GHz 8-core Central Processing Unit (CPU); 64.0 Gb physical memory) running Microsoft Windows 10. An Orion HD frame grabber (Matrox Imaging, Montreal, CA) captured and stored uncompressed video from the video processor. Image processing was offloaded to an Nvidia TITAN Xp graphics processing unit (GPU) installed within the workstation. The system also included a portable monitor for displaying a post-processed computational video feed that was visualized by the research team. Communication with the light modulation controller and direct drive rotation mount occurred over native universal serial bus (USB).

**Data acquisition software:** A custom C++ application was developed to enable real-time data acquisition and control of the MLE system. Independent application threads were launched for (1) a command line interface (CLI) to receive user inputs for transitioning between MLE illumination modes, (2) asynchronously processing data received by the frame grabber and updating the displays, and (3) communicating with the light modulation controller.

Multiple buffering was employed to enable concurrent frame capturing and processing. Frames were continuously acquired by the frame grabber and transmitted to the host desktop, where they were sequentially stored in a list of image buffers pre-allocated in physical memory. In parallel, the frame processing thread sequentially copied frames from the image buffer list and processed them, freeing spots in it. By continuously grabbing and storing in a list of image buffers, the system was robust to changes in operating system overhead, preventing frames from being dropped as long as the list was never filled. Once copied by the frame processing thread, the frame was written to the workstation's solid-state drive in an uncompressed format. The frame was then copied to GPU memory for image processing.

Image processing was executed on the GPU for accelerated computation. First, each frame was deinterlaced by separating the odd and even fields into separate arrays. The fields were then resized to the original image height using linear interpolation. The deinterlaced frames were processed and rendered to Display Monitor 1 and Display Monitor 2. Next, the mean intensity of each color channel for each deinterlaced frame was computed using a highly parallelized sum reduction algorithm implemented using the Compute Unified Device Architecture (CUDA) platform. If a cap attachment was used with the colonoscope, a circular mask was applied to exclude pixels occluded by the cap from the computed image statistics.

To maintain a well-exposed image with changes in working distance and tissue reflectance, the computed mean intensity values were used to generate updated pulse width lengths for every diode. Pulse width updates were computed using an auto-exposure technique based on an adaptation of the secant root-solving method. The algorithm assumes that image brightness and exposure time (pulse width length) are related by an unknown, monotonically increasing function. Convergence towards the function's root (the target mean intensity) is achieved by iteratively approximating the function with a secant line and solving for the line's root. This technique has several advantages, including fast convergence when applied to nonlinear functions (such as gamma-corrected images) and the ability to recover from erroneous values (caused by light modulation controller malfunctions, for example).

Given a mean intensity value $I_n$ illuminated with pulse width length $P_n$, an updated pulse width length $P_{n+1}$ can be computed using

$$P_{n+1} = \frac{[I_{\max} - I_n] \cdot P_n \cdot P_{\max}}{[I_{\text{target}} - I_n] \cdot P_n + [I_{\max} - I_{\text{target}}] \cdot P_{\max}}, \quad (1)$$

where $I_{\max}$ is the upper bound of the mean intensity (255), $P_{\max}$ is the maximum pulse width length (14 ms), and $I_{\text{target}}$ is the target mean image intensity (128). Pulse widths were restricted to a maximum length of 14 ms to avoid bleeding into the next image field acquired by the colonoscope. $I_n$ was set to the mean intensity value from the color channel with the highest Bayer transmissivity for the laser diode being updated. For white light illumination, the mean intensity values for all three color channels were averaged to generate a single value. While imaging with light from the high coherence laser, pulse width lengths were linearly mapped to angles formed by the fast axis of the half wave plate and the vertical axis of the laser (0 ms $\rightarrow$ 0°, 14 ms $\rightarrow$ 45°). Updated pulse width lengths and half-wave plate rotation angles were continuously written to a log file to record all illumination powers used for each frame.

Updated pulse width lengths for the odd and even fields were sent to the light modulation controller in 64 byte packets containing a unique frame ID (4 bytes), 16 bit pulse width lengths for each diode for the odd field (30 bytes), and 16 bit pulse width lengths for each diode for the even field (30 bytes). Measured laser output powers were sent from the light modulation controller to the host in 16 byte packets containing the received frame ID (4 bytes), 16 bit power measurements for the odd field (6 bytes), 16 bit power measurements for the even field (6 bytes). First-in-first-out (FIFO) buffers were pre-allocated on the host and the MCU for sending/receiving packets asynchronously to prevent blocking. Upon connection with the host desktop, the light modulation controller pulse width buffer was populated with a set of empty packets equal to the length of the image buffer list on the host desktop, effectively causing it to work several frames behind the host desktop. This ensured that the light modulation controller would not run out of pulse width lengths even when the host desktop fell behind on frame processing. An image buffer list 10 frames in length was empirically determined to provide an optimal balance between preventing dropped frames and minimizing latency in auto-exposure response.

Upon sending a pulse width packet to the light modulation controller, several frame acquisitions occur before the frame illuminated by the pulse width packet is seen and processed by the frame processing thread. This delay depends on the length of the pulse width buffer, the delay between frame acquisition and output by the video processor, and the delay in frame acquisition by the video capture card. Equation 1 accepts pairs of pulse width lengths and corresponding image mean intensities, so the delay between sending a pulse width and processing the corresponding image on the host desktop must be determined. To measure the delay, a single illumination synchronization pulse was sent to the light modulation controller. A counter was incremented with each frame processed by the frame processing thread, terminating once the frame containing the synchronization pulse was detected. The delay length varied between clinical systems, so a new measurement was taken during each imaging session. The delay was typically 23-24 frames, resulting in an auto-exposure response delay equal to approximately 0.8 s.

# 5 Supplementary videos

**Supplementary video 1:** Total oxygen saturation measurements of an index finger during an occlusion trial captured using MLE. (`https://youtu.be/2bmZE6es6qk`)

**Supplementary video 2:** Laser speckle contrast imaging of vasculature in the soft palate captured using MLE in freehand operation. (`https://youtu.be/n81Og6s7ksA`)

**Supplementary video 3:** Topographic imaging of the ventral tongue captured using MLE in freehand operation. (`https://youtu.be/qbiMa24wXTI`)

**Supplementary video 4:** 4 mm tubular adenoma in the ascending colon imaged *in vivo* with WLE and MLE's topographic, blood flow, oxygenation, and spectral enhancement (with unsharp masking) modes. (`https://youtu.be/iw3IiS3qWt0`)

**Supplementary video 5:** 5 mm tubular adenoma in the descending colon imaged *in vivo* with MLE's topographic, blood flow, oxygenation, and spectral enhancement (with unsharp masking) modes. (`https://youtu.be/3UQOom2GAHo`)

**Supplementary video 6:** 3 mm tubular adenoma in the cecum imaged *in vivo* with MLE's topographic enhancement mode. (`https://youtu.be/YRjAWZGeLS8`)

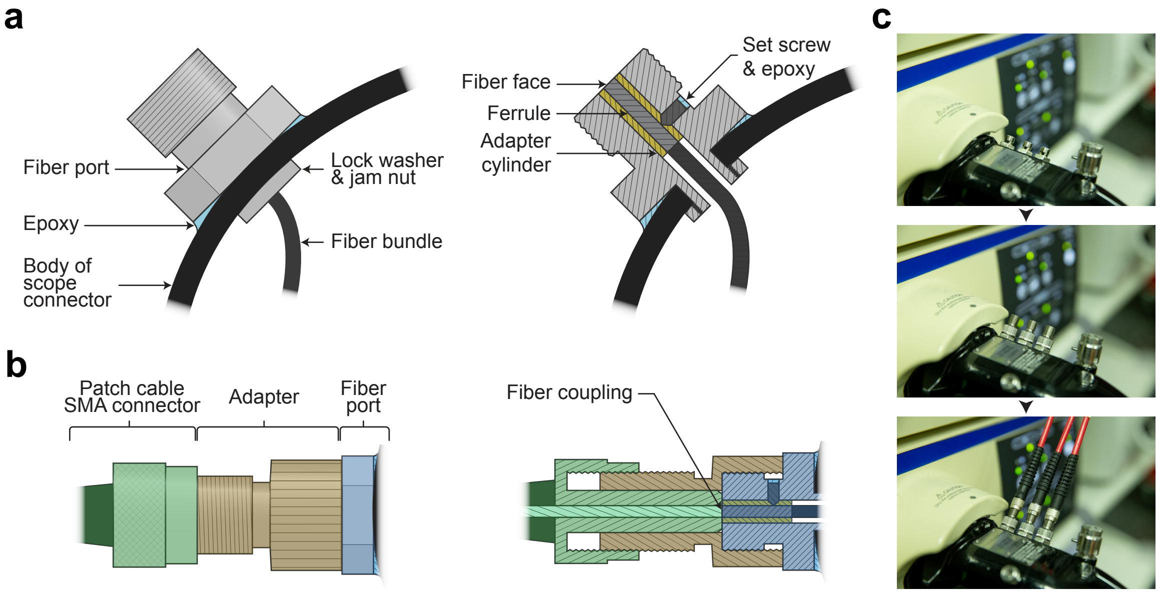


**Supplementary Figure 1: Scope connector modifications. a** Three externally threaded SMA fiber ports were installed through the connector end of the colonoscope. The MLE fiber bundle ferrules were passed from the scope's internal lumen through the fiber ports and secured. External surfaces were smoothed with epoxy to minimize crevices that could harbor bacteria. **b** Custom internal-to-external SMA-threaded adapters were machined for coupling SMA-terminated fiber patch cables to the MLE fiber bundles. **c** Images of the MLE fiber ports, SMA-threaded adapters, and fiber patch cables.

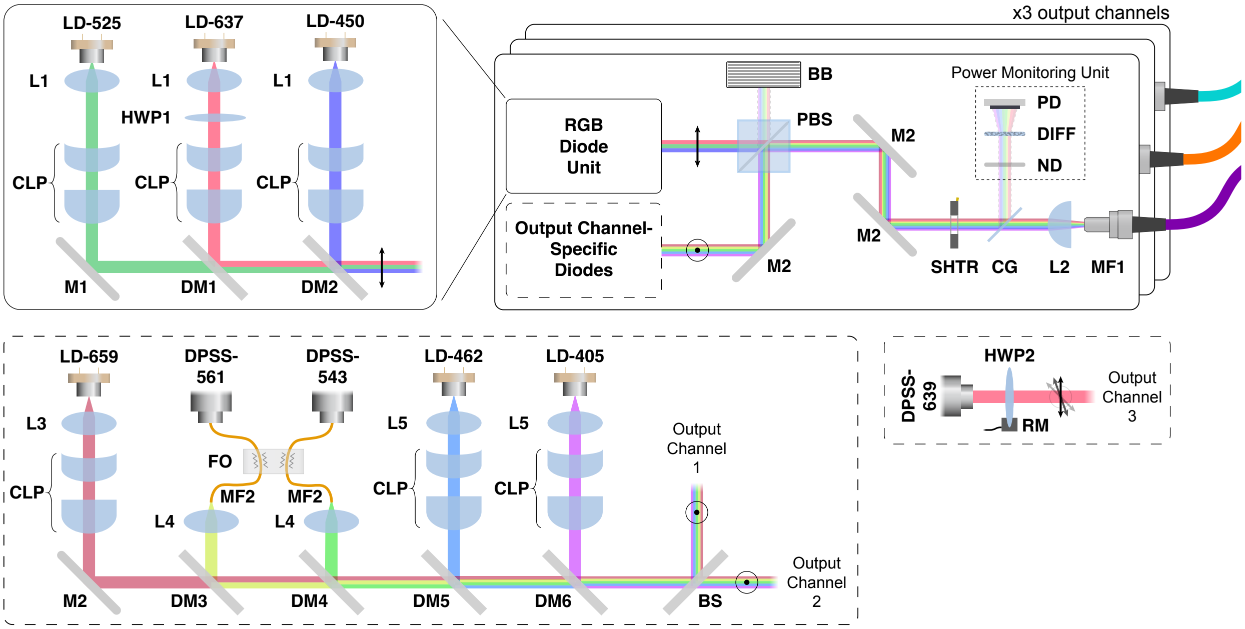


**Supplementary Figure 2: Laser illumination source optical schematic.** Three RGB laser units are used for white light and directional illumination modes. An additional 5 wavelengths are coupled into output channels 1 and 2 to expand the diversity of wavelengths available for multispectral imaging. A high-coherence laser is coupled into output channel 3 for speckle illumination. BB: Beam Block, BS: Beam Splitter, CG: Glass Coverslip, CLP: Cylindrical Lens Pair, DIFF: Diffuser, DM: Dichroic Mirror, DPSS: Diode-Pumped Solid-State Laser, FO: Fiber Oscillator, HWP: Half-Wave Plate, L: Aspheric Lens, LD: Laser Diode, M: Mirror, MF: Multimode Fiber, ND: Neutral Density Filter, PBS: Polarized Beam Splitter, PD: Photodiode, RM: Rotation Mount, SHTR: Shutter. A detailed component list is provided in Supplementary Table 1.

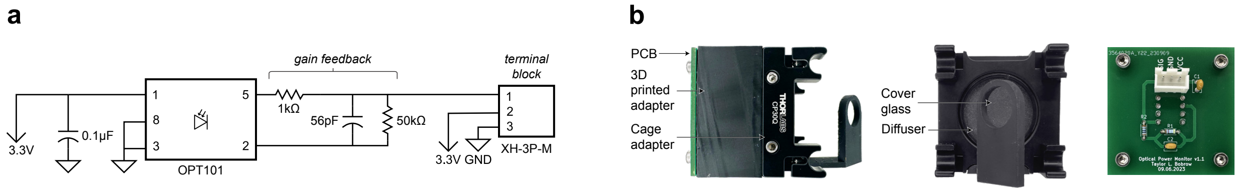


**Supplementary Figure 3: Laser diode output power monitoring unit.** (a) Electrical schematic. (b) Annotated photographs of the unit assembly. A cover glass slide picks off a small fraction of light for measurement by the photodiode. A ground glass diffuser is mounted between the coverglass and the photodiode to reduce the unit's sensitivity to misalignment.

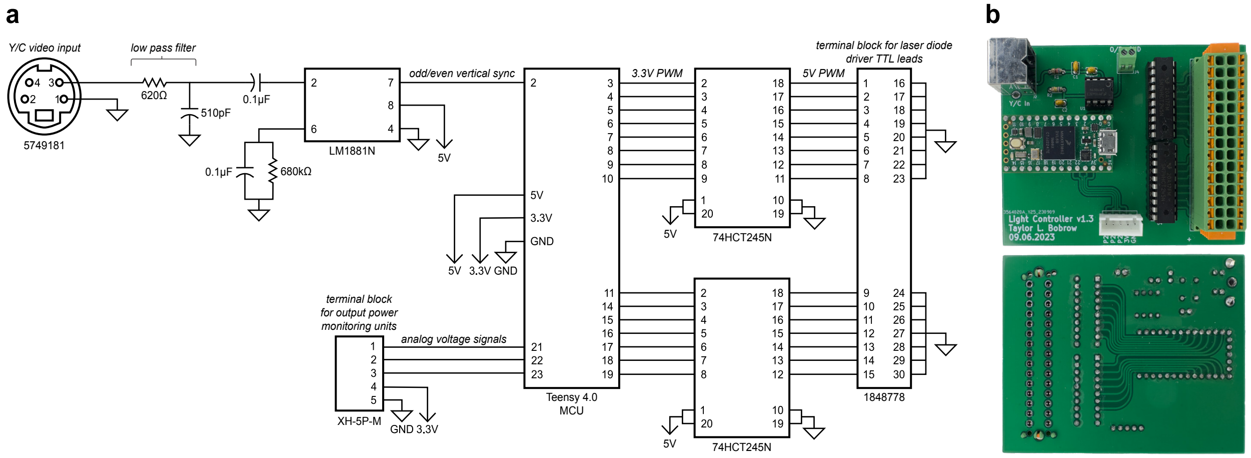


**Supplementary Figure 4: Light modulation controller.** **a** Electrical schematic. **b** Front (top) and back (bottom) photographs of the custom-fabricated PCB.

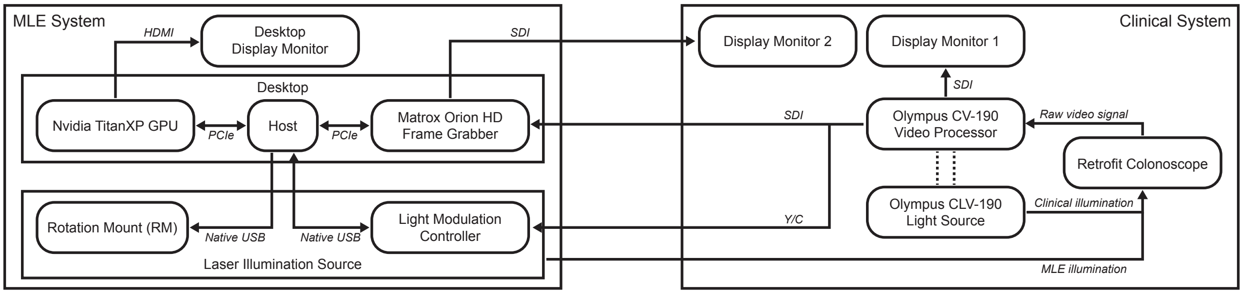


**Supplementary Figure 5: Wireframe diagram of MLE and clinical system architecture.**

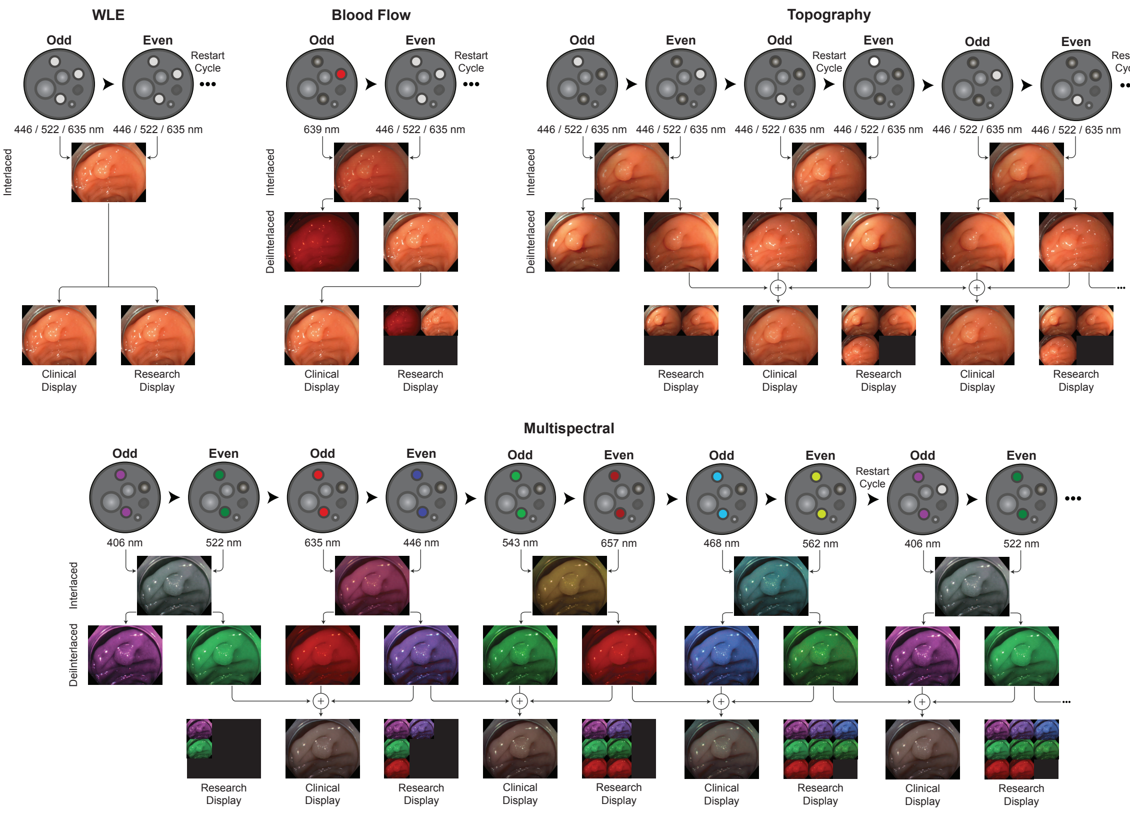


**Supplementary Figure 6: MLE illumination modes.** The MLE system was pre-programmed to cycle between white light, topographic, laser speckle, and multispectral illumination modes. Each mode was defined with a laser diode sequence, relative diode intensities, color channels used for auto-exposure, and post-processing steps for research and clinical video display outputs.

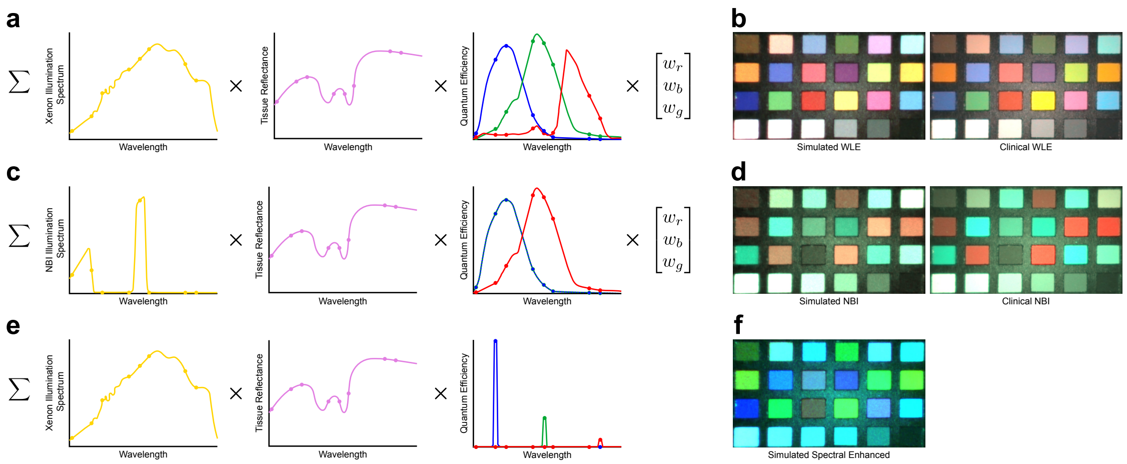


**Supplementary Figure 7: Color image simulation.** **a** White light endoscopy (WLE) color simulation process. The xenon illumination spectrum from the Olympus CLV-190 light source is attenuated by reflectance spectra acquired with MLE and the transmission spectrum of a commercial Bayer camera filter. A correction factor ($\omega$) is applied to each color channel to compensate for differences in the total energy between the complete and sparsely sampled illumination/camera transmissivity. The correction factor is computed by dividing the sum of the product of the full illumination spectra and color channel by the eight sparsely sampled spectra. **b** Ground truth and simulated WLE images. The simulated image was linearly color balanced using the ground truth image as a reference. **c** Narrow band imaging (NBI) color simulation process. The NBI illumination spectrum from the Olympus CLV-190 light source is attenuated by reflectance spectra acquired with MLE and the NBI color channel weightings. Intensity values from the blue Bayer channel are assigned to the green/blue image channels, and intensity values from the green Bayer channel are assigned to the red image channel. **d** Ground truth and simulated NBI images. The simulated image was linearly color balanced using the ground truth image as a reference. **e** Spectral enhanced color simulation process. The xenon illumination spectrum is attenuated by reflectance spectra acquired with MLE and camera transmission spectra obtained from the optimizer. **f** Simulated spectral enhanced image.

**Supplementary Table 1:** Laser illumination source optical components

| Key | Component |
|---|---|
| BB | Beam Block (LB1; Thorlabs, Inc., Newton, NJ, US) |
| BS | 50:50 UVFS Plate Beam Splitter (BSW26; Thorlabs, Inc., Newton, NJ, US) |
| CG | Glass Coverslip, $\varnothing$ = 12 mm (26023; Ted Pella Inc., Redding, CA, US) |
| CLP | Cylindrical Lens Pair, M = 3× (001024; Opt Lasers, Warszawa, PL) |
| DIFF | Ground Glass Diffuser, 120 Grit (DG10-120; Thorlabs, Inc., Newton, NJ, US) |
| DM1 | SP Dichroic Mirror (001410; Opt Lasers, Warszawa, PL) |
| DM2 | LP Dichroic Mirror (000424; Opt Lasers, Warszawa, PL) |
| DM3 | LP Dichroic Mirror, $\lambda_{on}$ = 613 nm (86-394; Edmund Optics, Barrington, NJ, US) |
| DM4 | LP Dichroic Mirror, $\lambda_{on}$ = 552 nm (86-393; Edmund Optics, Barrington, NJ, US) |
| DM5 | LP Dichroic Mirror, $\lambda_{on}$ = 480 nm (86-391; Edmund Optics, Barrington, NJ, US) |
| DM6 | LP Dichroic Mirror, $\lambda_{on}$ = 427 nm (86-389; Edmund Optics, Barrington, NJ, US) |
| DPSS-543 | DPSS Laser, 543 nm, 1 W (MGL-FN-543-1W; CNI Laser, Changchun, CN) |
| DPSS-561 | DPSS Laser, 561 nm, 1 W (MGL-FN-561-1W; CNI Laser, Changchun, CN) |
| DPSS-639 | DPSS Laser, 639 nm, 300 mW (MSL-FN-639; CNI Laser, Changchun, CN) |
| FO | Fiber Oscillator (CNI Laser, Changchun, CN) |
| HWP1 | Wave Rotator Plate (001832; Opt Lasers, Warszawa, PL) |
| HWP2 | Half-Wave Plate (AHWP05M-580; Thorlabs, Inc., Newton, NJ, US) |
| L1 | Aspheric Lens, $f$ = 4.02 mm (001046; Opt Lasers, Warszawa, PL) |
| L2 | Aspheric Lens, $f$ = 8.00 mm (A240TM-A; Thorlabs, Inc., Newton, NJ, US) |
| L3 | Aspheric Lens, $f$ = 4.51 mm (C230TMD-A; Thorlabs, Inc., Newton, NJ, US) |
| L4 | Fiber Collimator, $f$ = 10.92 mm (F220SMA-A; Thorlabs, Inc., Newton, NJ, US) |
| L5 | Aspheric Lens, $f$ = 3.10 mm (C330TMD-A; Thorlabs, Inc., Newton, NJ, US) |
| LD-405 | MM Laser Diode, 405 nm, 1 W (HL40033G; Ushio, Inc., Tokyo, JP) |
| LD-450 | MM Laser Diode, 450 nm, 1.6 W (PLTB450B; Osram, Munich, DE) |
| LD-462 | MM Laser Diode, 462 nm, 1.4 W (NDB7675; Nichia Co., Tokushima, JP) |
| LD-525 | MM Laser Diode, 525 nm, 1 W (NDG7475; Nichia Co., Tokushima, JP) |
| LD-637 | MM Laser Diode, 637 nm, 1.2 W (HL63283HD; Ushio, Inc., Tokyo, JP) |
| LD-659 | MM Laser Diode, 659 nm, 1.2 W (HL65213HD; Ushio, Inc., Tokyo, JP) |
| M1 | RGB Mirror (000874; Opt Lasers, Warszawa, PL) |
| M2 | Broadband Dielectric Mirror (BB1-E02; Thorlabs, Inc., Newton, NJ, US) |
| MF1 | MM Fiber, $\varnothing$ = 1 mm, L = 2 m (M59L02; Thorlabs, Inc., Newton, NJ, US) |
| MF2 | MM Fiber, $\varnothing$ = 100 µm, L = 5 m (M59L02; Thorlabs, Inc., Newton, NJ, US) |
| ND | Neutral Density Filter, OD = 0.8 (NE08A-A; Thorlabs, Inc., Newton, NJ, US) |
| PBS | Polarized Beam Splitter Cube (PBS; Opt Lasers, Warszawa, PL) |
| PD | Photodiode (OPT101; Texas Instruments, Dallas, TX, US) |
| RM | Direct Drive Rotation Mount (DDR25; Thorlabs, Inc., Newton, NJ, US) |
| SHTR | Beam Shutter (SM1SH1; Thorlabs, Inc., Newton, NJ, US) |

Abbreviations: Shortpass (SP), Longpass (LP), Multimode (MM)

**Supplementary Table 2:** Laser diode packages, drivers, and output powers

| **Key** | **Package** | **Peak $\lambda$ (nm)** | **Driver** | **Power (mW)*** |
|---|---|---|---|---|
| LD-405 | HL40033G | 406 | LPLDD-5A-24V-TP-H | 30 |
| LD-450 | PLTB450B | 446 | LPLDD-5A-24V-TP-H | 15 |
| LD-462 | NDB7675 | 468 | LPLDD-5A-24V-TP-H | 25 |
| LD-525 | NDG7475 | 522 | LPLDD-5A-24V-TP-H | 15 |
| DPSS-543 | MGL-FN-543 | 543 | PSU-H-LED | 25 |
| DPSS-561 | MGL-FN-561 | 562 | PSU-H-LED | 25 |
| LD-637 | HL63283HD | 635 | LPLDD-5A-24V-TP-H | 15 |
| DPSS-639 | MSL-FN-639 | 639 | PSU-H-FDA | 50 |
| LD-659 | HL65213HD | 657 | LPLDD-5A-24V-TP-H | 30 |

* Output power emitted by a single diode, measured at the distal tip of the colonoscope

**Supplementary Table 3:** *In vivo* colonoscopy study data summary

| **Patient #** | **Sample #** | **Histology** | **WLE** | **NBI** | **PSE** | **LSCI** | **Multispectral** |
|---|---|---|---|---|---|---|---|
| P1 | S1 | Tubular | ✓ | | ✓ | | |
| P2 | S1 | Serrated | ✓ | | ✓ | | |
| | S2 | Serrated | ✓ | | ✓ | | |
| P3 | S1 | Serrated | ✓ | | ✓ | ✓ | |
| P4 | S1 | Tubular | ✓ | | ✓ | | |
| | S2 | Tubular | ✓ | | ✓ | ✓ | |
| | S3 | Tubular | ✓ | ✓ | ✓ | ✓ | |
| P5 | S1 | Tubular | ✓ | | ✓ | ✓ | |
| P6 | S1 | Tubular | ✓ | | ✓ | ✓ | |
| P7 | S1 | Serrated | ✓ | ✓ | ✓ | | |
| P8 | S1 | Serrated | ✓ | ✓ | ✓ | | |
| P9 | S1 | Tubular | ✓ | | ✓ | ✓ | |
| P10 | S1 | Tubular | ✓ | | ✓ | ✓ | |
| P11 | S1 | Tubulovillous | ✓ | ✓ | ✓ | ✓ | |
| | S2 | Tubular | ✓ | | ✓ | ✓ | |
| P12 | S1 | Tubular | ✓ | ✓ | ✓ | | |
| P13 | S1 | Tubular | ✓ | ✓ | ✓ | ✓ | |
| | S2 | Tubular | ✓ | | ✓ | | |
| P14 | S1 | Serrated | ✓ | ✓ | ✓ | ✓ | ✓ |
| | S2 | Serrated | ✓ | ✓ | ✓ | | ✓ |
| P15 | S1 | Tubular | ✓ | ✓ | ✓ | ✓ | |
| | S2 | Tubular | ✓ | ✓ | ✓ | ✓ | |
| P16 | S1 | Tubular | ✓ | ✓ | ✓ | | |
| | S2 | Tubular | ✓ | ✓ | ✓ | ✓ | ✓ |
| P17 | S1 | Tubular | ✓ | ✓ | ✓ | ✓ | ✓ |
| P18 | S1 | Tubular | ✓ | ✓ | ✓ | ✓ | ✓ |
| | S2 | Tubular | ✓ | ✓ | ✓ | ✓ | ✓ |
| P19 | S1 | Tubular | ✓ | ✓ | ✓ | ✓ | ✓ |
| | S2 | Tubular | ✓ | ✓ | ✓ | ✓ | ✓ |
| P20 | S1 | Tubular | ✓ | ✓ | ✓ | ✓ | ✓ |
| | S2 | Tubular | ✓ | ✓ | ✓ | ✓ | ✓ |
